# Decitabine-based chemotherapy followed by haploidentical lymphocyte infusion improves the effectiveness in elderly patients with acute myeloid leukemia

**DOI:** 10.18632/oncotarget.11183

**Published:** 2016-08-10

**Authors:** Yu Jing, Xiangshu Jin, Lixin Wang, Liping Dou, Quanshun Wang, Yushi Yao, Shimei Lian, Jihao Zhou, Haiyan Zhu, Zilong Yao, Lijun Gao, Lili Wang, Yonghui Li, Xuefeng Bai, Meiyun Fang, Li Yu

**Affiliations:** ^1^ Department of Hematology, Chinese PLA General Hospital, Beijing, China; ^2^ Department of Hematology, Navy General Hospital of PLA, Beijing, China; ^3^ Department of Hematology, Dalian Municipal Central Hospital, Affiliated Hospital of Dalian Medical University, Dalian, China; ^4^ Department of Pathology and Comprehensive Cancer Center, The Ohio State University Medical Center, Columbus, OH, USA; ^5^ Department of Hematology, The First Affiliated Hospital of Dalian Medical University, Dalian, China

**Keywords:** decitabine, haploidentical lymphocyte infusion, Induction therapy, acute myeloid leukemia

## Abstract

In this study, we first initiated a multicenter, single-arm, phase-II clinical trial using decitabine (DAC) (20mg/m^2^ for five days) based chemotherapy, followed by haploidentical lymphocyte infusion (HLI) that was applied as induction therapy for elderly patients with AML. Furthermore, the role of HLI infusion was explored in a mouse model. The clinical trial included 29 elderly patients (median age: 64, range 57-77) with AML. Sixteen cases achieved complete remission (CR) and 9 cases achieved partial remission (PR) after the first treatment cycle. Of the patients with PR, 5 subjects achieved remission after the second induction, which brings the overall CR rate to 72.4%. The 2-year overall survival (OS) and disease-free survival (DFS) was 59.6% and 36.9% respectively. The treatment regimen was well tolerated with only one patient died of severe pneumonia one month after the first treatment. In the mouse experiment, we found that DAC/HLI significantly enhanced the survival of leukemic mice. These results suggested that DAC-based chemotherapy combined with HLI is an alternative first line induction therapy for elderly patients with AML. This trial is registered at ClinicalTrials.gov (NCT01690507).

## INTRODUCTION

The majority of acute myeloid leukemia (AML) patients are older individual who are usually deemed ineligible for intensive chemotherapy. Up until now, the outcome of conventional induction chemotherapy for older patients with AML has been unsatisfactory. The two years OS in this group of patients is less than 10 percent [1]. Decitabine (DAC), a demethylating agent, was approved by European Union for the treatment of elderly patients with newly diagnosed AML who are not eligible for standard chemotherapy. As an epigenetic modulator, low dose DAC provides a promising approach for AML. However, less than 50 percent of older patients with AML achieved CR when DAC was administrated alone [2, 3]. Recent studies showed that DAC can increase the chemosensitivity of leukemic [4] and several solid tumor cells [5], [6], [7]. Accumulating evidence also revealed that DAC may enhance the immunogenicity of malignant cells by inducing the expression of several immune-related genes including cancer testis antigen [8], major histocompatibility complex (MHC) molecules [9], costimulatory molecules [10] and adhesive molecules in these cells. Our previous study has demonstrated that DAC is capable of eliciting an anti-tumor CTL response by inducing CD80 expression in leukemic cells [11]. These two immunological effects of DAC offer it a great potential for developing DAC-based chemotherapy. However, the anti-leukemia activities of DAC-based chemotherapy could be limited by the immunocompromised status of AML patients due to chemotherapy and aging. Infusion of allogeneic lymphocytes following DAC-based therapy thus has the potential to overcome this limitation. Therefore, we hypothesized that adoptive transfer of haploidentical lymphocytes may act in synergy with DAC-based chemotherapy for AML.

In this study, we first initiated a multicenter, single-arm, phase-II clinical trial using DAC (20mg/m^2^ for five days)-based chemotherapy followed by infusion of haploidentical lymphocytes infusion (HLI) for the treatment of 29 elderly patients with AML. We found that DAC/HLI therapy was well tolerated and induced CR in 72.4% of AML patients. In an AML mice model, we further explored the role of HLI in the DAC/HLI combined chemotherapy. We found that DAC/HLI combination significantly enhanced the survival of leukemic mice. These results suggest that DAC-based chemotherapy combined with HLI is an alternative first line induction therapy for elderly patients with AML.

## RESULTS

### HLI enhanced the survival of patients with leukemia receiving DAC-based chemotherapy

DAC treatment causes cancer cells to express more tumor antigens and co-stimulatory molecules, thereby renders these cells more susceptible to T cell therapy. On the other hand, HLI has also been shown to be a promising method for the treatment of human leukemia [12]. Thus in this study, we designed a clinical trial protocol involving the use of DAC-based chemotherapy followed by HLI (ClinicalTrials.gov; NCT01690507). AML patients were assigned to receive chemotherapy with DAC (20 mg/m^2^ intravenously for 5 days), aclacinomycin (ACM, 20mg every second day intravenously for 5 days), cytarabine (10 mg/m^2^ every 12 hours subcutaneously for 5 days), granulocyte colony-stimulating factor (G-CSF, 300 μg/day subcutaneously from day 0 to neutrophil recovery), in combination with HLI treatment. HLI were performed 36 hours after the last dose of chemotherapy. Subjects who experienced CR or PR received a total of four repeated treatments. No maintenance treatment was given to patients who finished four treatments.

#### Patient characteristics

There were 29 patients with previously untreated AML defined by World Health Organization(WHO) criteria enrolled in the study, including 10 (34.5%) women and 19 (65.5%) men. The basic information of these patients is shown in Table 1. The median age of these patients was 64 (range 57-77), with 5 patients less than 60, 14 patients with an age range of 60-69, and 6 patients over 70, 8 subjects (28%) had normal karyotype, 6 subjects (30%) complex karyotype, and 15 other cytogenetic abnormalities.

**Table 1:** Patient characteristics

	*n* (%)
Gender	
Men	19 (65.5)
Women	10 (34.5)
Diagnosis(WHO)	
M1	0 (0)
M2	16 (55.2)
M4	4 (13.8)
M5	7 (24.1)
M6	2 (6.9)
Age(years)	
55-59	5 (17.2)
60-64	14 (48.3)
65-69	4 (13.8)
≥70	6 (20.7)
Performance status(ECOG sore)	
1	5 (17.2)
2	12 (41.4)
3	12 (41.4)
WBC at diagnosis,10×10^9^/L	
≤20	22 (75.9)
>20-100	4 (13.8)
≥100	3 (10.3)
Cytogenetic risk	
Favorable	0 (0)
Intermediate	20 (69)
Unfavorable	7 (24.1)
unavilable	2 (6.9)
Comorbid conditions	
Lung Infection	4 (13.8)
Diabetes	6 (20.7)
Hypertension	12 (41.4)
Cardiovascular disease	6 (20.7)
Others	10 (34.5)

According to the validated Medical Research Council (MRC) prognostic risk score for clinical outcome in older AML patients [13], 27.5% of enrollees were in poor risk, 41.5% in standard risk, and 31% in good risk.

Twenty four subjects (82.75%) had a performance status of ≥2. The median white blood cell(WBC) count was 25.7×10^9^/L (range 0.1-270×10^9^/L). The median bone marrow blast count was 60% (20.6%-95%). Twenty two subjects had an intermediate and seven subjects (24%) were with an unfavorable cytogenetic risk. Two subjects (5%) had the MLL/MLL or MLL/AF9 fusion gene and three subjects (10%) had a WBC more than 100×10^9^/L. Seven subjects received cytarabine treatment due to the fact that their WBC were more than 20×10^9^/L before the initiation of DAC-based chemotherapy. Cytarabine was given to the patients at 100mg to 200mg intravenously each day, and was stopped when WBC was lower than 10×10^9^/L. The DAC/HLI treatment was started at the next day after the cessation of cytarabine. The median follow-up was 18.2 months (range 2-33.7 months).

#### Efficacy of HLI and DAC-based combination therapy

Of 29 patients receiving combination treatment with DAC-based chemotherapy and HLI, 16 achieved CR and 9 achieved partial PR after the first treatment cycle. Of the patients with PR, 5 subjects achieved CR after the second induction, which brings the overall CR rate to 72.4%. CR occurred in all risk groups. For subjects with good risk, 88.9% (8 of 9) achieved CR. For subjects with standard risk, 66.7% (8 of 12) achieved CR. For subjects with poor risk, 62.5% (5 of 8) achieved CR.

**Figure 1:**
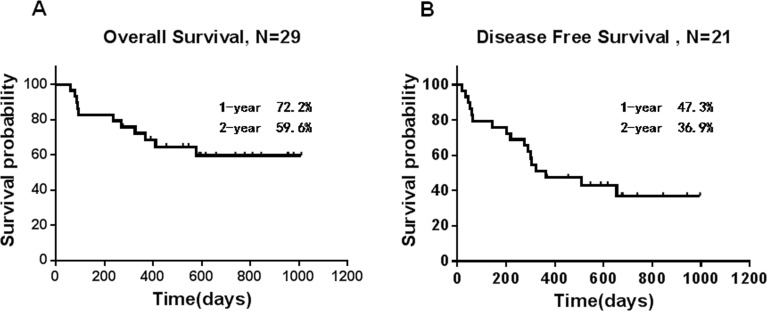
Survival results **A.** Overall survival and **B.** disease-free survival. Vertical marks reflect last follow-up times for censored observations.

Upon final analysis (May 31, 2015), the median survival time was 546 days for all 29 subjects. The 2-year probability of OS (Figure 1A) and Disease-free survival (DFS) (Figure 1B) was 59.6±9.6% and 36.9±9.9%, respectively. The 1-year probability of OS and DFS of patients received prior cytarabine therapy due to hyperleukocytosis were 42.9±18.7% and 14.3±13.2%, respectively. For patients with no prior therapy, The 1-year probability of OS and DFS was 76.4±9.3% and 50.9±11.3%, respectively. For subjects with good risk, the 1-year probability of OS and DFS was 64.8±16.5% and 41.5±20.7%, respectively. For subjects with standard risk, the 1-year probability of OS and DFS was 83.3±10.8% and 58.3±14.2%, respectively. For subjects with poor risk, the 1-year probability of OS and DFS was 37.5%±17.1% and 25±15.3%, respectively. 21 patients achieved CR after two cycle of treatment, the 1-year probability of OS and DFS was 80.4±8.8% and 54.5±11.3%, respectively. There were only 2 patients who achieved partial remission(PR) after two cycle of treatment. One patient refused further treatment and died within 90 days. The other patient received best supportive care and traditional Chinese medicine treatment and died 411 days after diagnosis. There were limited data about the patients with molecular markers. One patient with FLT3-ITD did not achieve CR and died of disease progress within 3 months. One patient with MLL/AF9 achieved molecular remission after 2 cycles of treatment and received 2 cycles of consolidation treatment with the same scheme of induction. The patient relapsed 4 months after the last treatment. She could not achieve CR and died within 3 months. One patient with MLL/MLL achieved morphologic CR and the ratio of fusion gene/ABL decreased from 26.3% to 4.3% after 2 cycle of treatment. He received additional 2 cycle of consolidation treatment with the same regimen but failed to test the fusion gene. He relapsed 6 months later and received chemotherapy with mitoxantrone and cytarabine. CR was achieved again. He remained CR up until the last follow-up.

Thirteen subjects (44.8%) died during the follow-up. Of those, three subjects did not achieve CR and died of disease progression and 6 subjects died of leukemia relapse, 2 subjects died of pneumonia, including one patient died at 37 days after chemotherapy, and 2 subjects died of gastrointestinal hemorrhage.

#### Safety and toxicity

**Figure 2:**
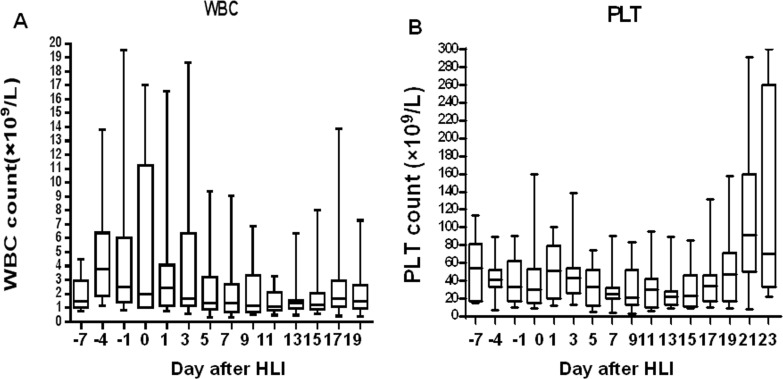
Blood count recovery after induction therapy of DAC-based chemotherapy combined with haplo-identical donor lymphocytes Box plots show the median (line), interquartile (box extents), and range (whiskers) of WBC **A.** and platelet count **B.** for all patients during the time of the induction therapy. Blood count data are summarized every second day.

Overall, the combination regimen was well tolerated. The median recovery times for neutrophils and platelets were 11 days and 14 days, respectively, after the first cycle of chemotherapy. The median number of WBC and platelets were more than 1.0×10^9^/L (Figure 2A) and 20×10^9^/L (Figure 2B), respectively, after the first cycle of chemotherapy induction. Three patients did not exhibit neutropenia. The platelet counts of 2 patients kept higher than 20×10^9^/L during the whole treatment. Myelosuppression was commonly observed, and febrile neutropenia occurred in 55.2% of patients. No subjects needed to be transferred to Intensive Care Unit.

All patients were evaluated for toxicity. The most common grade 3 or 4 adverse events (AE) are listed in Table 2. Infectious complications were rare following achievement of CR. Myelosuppression during maintenance treatment given after CR was minimal.

**Table 2:** Treatment-Emergent Grades III/IV AEs during the first two cycles

AEs of ≥Grade III	No.	Percentage
Atrial fibrilation	1/29	3.4%
Blood bilirubin increased	1/29	3.4%
Diarrhea	1/29	3.4%
Febrile neutropenia	16/29	55.2%
Gastrointestinal hemorrhage	1/29	3.4%
Heart failure	1/29	3.4%
Lung infection	2/29	6.8%
Rash	1/29	3.4%

#### GVHD and severe infection

No symptoms of acute or chronic Graft *Versus* Host Disease (GVHD), such as unexplained skin rashes, diarrhea, increased bilirubin or disturbance of coagulation, was observed in any of the subjects during treatment. Four subjects suffered from pneumonia during the first 2 cycles of chemotherapy. Two subjects were diagnosed as pulmonary fungal infection between the second and third cycle of chemotherapy and were controlled with anti-fungal agents.

### HLI enhanced the survival of mice with leukemia receiving DAC-based chemotherapy

**Figure 3:**
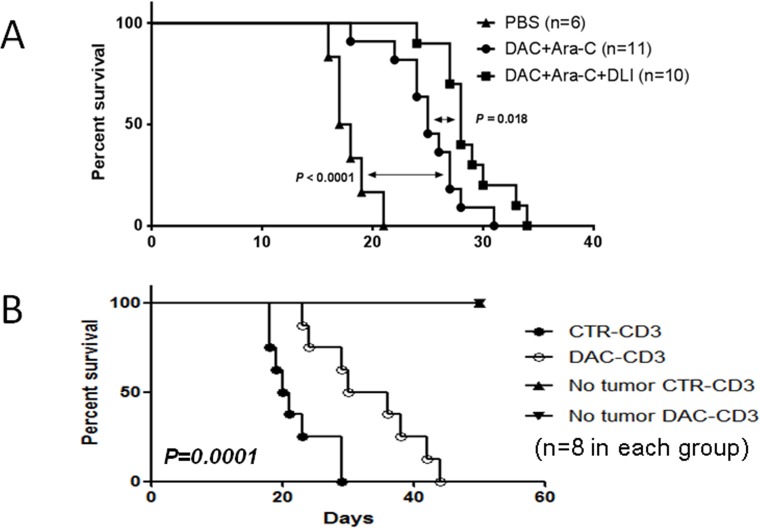
DAC treatment induced autologous anti-leukemia response and acted in synergy with adoptive T cells transfusion **A.** WEHI-3 cells were injected *i.p*. into Balb/c mice at 1×10^5^ cells/mouse. DAC (0.5mg/kg/day for 5 days) and cytarabine (10mg/kg/d for 5 days) were administrated 5 days later. Lymphocyte from CBF1 mice were injected intravenously 48 hours later. Mouse survival was monitored every two or three days. Five to six mice were used in each group and data were pooled from two experiments. **B.** WEHI-3 cells were injected *i.p*. into Balb/c mice at 1×10^5^ cells/mouse. DAC (1mg/kg/day for 3 days) or PBS were administrated 5 days later. Purified CD3^+^ cells were harvested from each mouse 7 days after the treatment for transfer. Meanwhile, *in vitro* DAC (0.25uM for 3 days) treated WEHI3 cells were injected into naïve Balb/c nude mice *i.p*. at 5×10^4^ cells/mouse. Then harvested CD3^+^ cells were adoptively transferred intravenously into with/without WEHI3 nude mice at 2×10^7^ cells/mouse. Mouse survival data is shown. Eight mice were included in each group and data shown are representative of two independent experiments.

The results of our clinical trial showed that the DAC-based chemotherapy combined with HLI achieved higher CR rate than that of intensive chemotherapy. We hypothesized that HLI may act synergistically with DAC-based chemotherapy in eradicating leukemic cells. To test this hypothesis, we conducted a series of experiments using a mouse AML model. BALB/c mice were injected with mouse leukemia WEHI-3 cells *i.p*. and treated with DAC and cytarabine(Ara-C) with or without HLI. As shown in Figure 3A, while DAC+Ara-C combination showed significant efficacy in promoting the survival of mice with leukemia, HLI further improved the survival of mice treated with DAC+Ara-C combination. These data suggested that HLI enhances the efficacy of DAC+Ara-C chemotherapy, leading to longer survival of mice with leukemia.

While the detailed mechanisms of HLI-mediated survival benefit remain elusive, We have previously demonstrated that DAC treatment induces anti-tumor CTL responses in a mouse solid tumor model. Herein we hypothesized that DAC treatment could render tumor cells more susceptible to the destruction induced by lymphocytes such as CTLs; Furthermore, DAC has the potential to enhance the priming of T cell responses, which may act in synergistic with adoptive transfer of HLI. To test the effect of DAC treatment on CTL response in mice with leukemia, BALB/c mice with leukemia were treated with PBS or DAC. Seven days later mice were sacrificed and spleen cells were harvested. CD3+ cells were purified using flow cytometry-based sorting followed by infusion into BALB/c nude mice. The nude mice were then challenged with DAC-treated WEHI-3 cells. As shown in Figure 3B, mice receiving T cells from DAC-treated mice experienced significantly longer survival compared with mice treated with T cells from control mice.

## DISCUSSION

In this study, we first initiated a multicenter, single-arm, phase-II clinical trial using DAC/HLI to treat 29 elderly patients with AML. We found that DAC/HLI therapy was well-tolerated and induced CR in a total of 72.4% of AML patients after two cycles of treatments. Notably, 6 out of 29 (20.6%) patients treated in this study were older than 70 years old, and 24 (86.7%) had ECOG Performance Status (ECOG PS) ≥2. Furthermore, we conducted a series of experiments to investigate the role of HLI in an AML mouse model. We found that mice received HLI containing therapy had a significant longer survival than mice received chemotherapy alone, which suggested HLI exerts a synergistic effect with DAC-based chemotherapy. In addition, we revealed DAC treatment elicited autologous CTL response against AML.

For the last two decades, little progress has been made in the treatment of elderly patients with AML and there remains no consensus regarding optimal therapy. The outcome of the intensive chemotherapy in elderly patients with AML has been unsatisfying. Generally, the CR rate was about 40-55% and the early death (ED) rate was 19-29% [14].

Low dose cytarabine, aclaribicin and G-CSF (CAG) regimen was originally designed in Japan for the treatment of relapsed AML patients [15]. Several clinical studies showed CAG regimen achieved about 50% CR in elderly patients with AML with lower early death(about 8%) [16, 17]. In a recent study, single-agent DAC was conducted in older patients (≥60 years) with previously untreated AML who were not candidates for or refused intensive chemotherapy. The complete remission rate was 47% (*n* = 25), achieved after a median of three cycles of therapy. Death within 8 weeks occurred in 15% of subjects [3]. The results of our clinical trial showed that DAC/HLI was well tolerated and effective with CR of 72.4% in elderly patients with AML after two cycle of treatment, while only 1 patient (3.4%) died within 8 weeks. A recent study reported a prospective phase II clinical trial assessing the safety and efficacy of D-CAG (DAC combined with cytarabine, aclarubicin, and G-CSF) induction treatment for elderly patients with newly diagnosed AML [18]. Among 85 evaluable patients, CR was 64.7% after 1 cycle of therapy. The induction mortality was 4.4%. According to their report, 77.6% patients achieved CR after two cycles of treatment. The CR rate of their regimen is comparable to the data presented here. While agents in their treatment were the same as ours, the duration and dosage of cytarabine and aclarubicin were different. The most significant difference between the two regimens was that HLI was included in ours at day seven. It is difficult to evaluate the role of HLI directly because of different conditions and settings in these two studies. Our animal experiments demonstrated that HLI exerted synergistic effects with previous DAC-based chemotherapy. Apparently, prospective, randomized and control studies are needed to investigate this aspect. In addition, we believed there still is a plenty of room for us to improve the combination treatment by optimizing the cell components.

In this clinical study, 7 patients received prior cytarabine due to the fact that there was a high number of circulating peripheral blasts or that they were even hyperleukocytair. Given cytarabine is the cornerstone of an AML induction therapy, we compared the OS and DFS of patients who received prior cytarabine therapy with those who did not receive prior therapy. We found that prior cytarabine therapy did not contribute to longer OS or DFS. On the other hand, hyperleukocytair might play a central role in the survival of these patients.

The results of our clinical trial demonstrated that the combination treatment was well tolerated for elderly patients, who were traditionally deemed as unfit intensive chemotherapy. There was only one patient died within the first 8 weeks. The degree of myelosuppresion was relatively lower than that after intensive regimen. The duration of neutropenia was only 11 days. We speculated that HLI may play an important role to enhance the immune recovery after chemotherapy and to reduce the risk of infections.

In the study, 6 out of 21 patients who achieved CR experienced relapse within median duration of 8.4 months(5.9-21.6 months). Among these 6 patients, 3 were in poor risk, 1 in standard risk, and 2 in good risk. For elderly patients with AML, there is no consensus post-remission treatment available [19]. Additional 2 or 3 cycles of treatment were given to the patients after they achieved CR in our study. The relapse rate suggested that more maintenance treatment should be considered for these patients.

In conclusion, our study demonstrated that DAC-based chemotherapy followed by HLI is a safe and effective induction regimen for untreated elderly patients with AML. The results suggested that DAC/HLI may significantly improve responding rate compared with other chemotherapy regimens those are currently using. Further a randomized-controlled trial in a large cohort of subjects is required to confirm the safety and efficacy of this combination therapy. The underlying therapeutic mechanisms, in particular the role of HLI, also warrant further investigations.

## MATERIALS AND METHODS

### Patients

Twenty nine patients with newly diagnosed AML (from April, 2012 to May, 2015) were enrolled in this study. The eligibility criteria were age over 55 years with previously untreated AML (marrow blasts ≥ 20%, *de novo* or secondary to myelodysplastic syndrome). The diagnoses were defined by the French-American-British and World Health Organization criteria. Chromosomal analysis and immunophenotyping were performed for all subjects using bone marrows obtained at diagnosis.

High resolution HLA-typing was conducted in all subjects and donors. Sex, age, and other characteristics of lymphocyte donors were not considered as a priority. Of the 29 patient/donor pairs, the median age of donors was 34 (range 24-54), including 15 sons, 12 daughters, 1 younger brothers and 1 younger sister.

The protocol was approved by the Human Ethics Committees of the Chinese PLA General Hospital, Beijing; Navy General Hospital of PLA, Beijing; the First Affilliated Hospital of Dalian Medical University, Liaoning province; and Dalian Municipal Central Hospital Affilliated to Dalian Medical University, Liaoning Province, China. This protocol was conducted in accordance with the Declaration of Helsinki. All patients and donors gave written informed consent before enrollment into the study. This trial was registered at www.clinicaltrials.gov as NCT01690507.

### Treatment design

Subjects were assigned to receive chemotherapy with DAC (20 mg/m^2^ intravenously for 5 days), aclacinomycin (ACM, 20mg every second day intravenously for 5 days), cytarabine (10 mg/m^2^ every 12 hours subcutaneously for 5 days), granulocyte colony-stimulating factor (G-CSF, 300 μg/day subcutaneously from day 0 to neutrophil recovery) every 4 weeks. The subsequent cycles was individually customized based on the duration of neutropenia. HLI were administered 36 hours after the final dose of chemotherapy. For the first induction therapy, the cytarabine was given to the patients with white blood cell(WBC) count higher than 20×10^9^/L at 100mg to 200mg intravenously for each day, and was stopped when WBC was lower than 10×10^9^/L. The DAC/HLI treatment was started at the next day after the cessation of cytarabine. Subjects who experienced CR or PR received a total of four courses of the same treatment. No maintenance treatment was given to patients who finished four treatments.

Routine blood counts, electrolyte levels, liver function tests, and creatinine levels were monitored twice a week. Adverse events, concomitant medications, and clinical laboratory analyses were recorded weekly. The treatment was discontinued in cases of disease progression, intolerable toxicity, death, losses of follow-up, treatment abandonment, or withdrawal of consent to further treatment. All subjects received supportive care, including blood product transfusions, prophylactic or symptomatic use of antibiotic agents and cytokines, according to institutional practices and other therapies appropriate for the symptomatic treatment for AML and its complications.

Subjects who did not achieve CR after two cycles of chemotherapy, or those who experienced relapses during the first two cycles were considered as treatment failure and treatment was discontinued. None of the subjects received any GVHD prophylactic treatment or further maintenance therapy.

### Apheresis of donor peripheral mononuclear cells

Apheresis of haplotype-identical donor peripheral mononuclear cells was carried out with a cell separator (COBE SPECTRA cell separator). Donor cells were divided into aliquots and were cryopreserved in liquid nitrogen, whereas freshly collected cells were used in the first cycle. Infusion was performed at 36 hours after each chemotherapy regimen. The median numbers of mononuclear cells, CD3^+^, CD3^−^CD19^+^, and CD3^−^CD16^+^CD56^+^ cells infused per treatment were 1.51×10^8^/kg (0.77-2.57×10^8^/kg), 0.935×10^8^/kg (0.62-2.03×10^8^/kg), 0.18×10^8^/kg (0.048-0.3×10^8^/kg) and 0.089×10^8^/kg (0.041-0.23×10^8^/kg), respectively.

### Response criteria and outcome evaluation

Routine blood cell counts were performed twice per week after chemotherapy. At three to four weeks after chemotherapy, a bone marrow aspiration was performed and responses to treatment were evaluated. Responses were determined according to the revised recommendations of the International Working Group (IWG) for Diagnosis, Standardization of Response Criteria, Treatment Outcomes, and Reporting Standards for Therapeutic Trials in Acute Myeloid Leukemia. OS was defined as the time from diagnosis to death or to the last date of follow-up until May 2015. For secondary end points, bone marrow biopsies and aspirates were obtained from subjects at screening.

The following conditions were defined according to the IWG criteria: (1) CR was diagnosed for subjects demonstrating less than 5% bone marrow blasts, no blasts with Auer rods, the absence of extramedullary disease, an absolute neutrophil count > 1×10^9^/L, and a platelet count ≥100×10^9^/L; (2) PR was assigned to subjects with a decrease of at least 50% (between 5 and 20%) in the frequency of blasts detected in bone marrow aspirates and those with normalized blood counts; (3) No remission(NR) was defined as a response that had achieved neither CR nor PR; and (4) relapse was defined as the reappearance of leukemia cells in the peripheral blood or the detection of more than 5% blasts in the bone marrow. Early death was defined as mortality within the first 8 weeks after induction of chemotherapy. GVHD was defined according to published criteria [20].

The recovery time of neutrophils and platelets was defined as the first of 3 consecutive days on which the absolute neutrophil count and platelet count exceeded 0.5×10^9^/L and 30×10^9^/L, respectively, since the day of HLI. Adverse events were determined according to the CTCAE version 4.03.

### Mice and cells

Balb/c and Balb/c nude mice (male, 6-8 week) were purchased from Beijing Vital-River Lab Animal Technology Co. Ltd. WEHI-3 (a mouse AML cell line) was originally obtained from American Type Culture Collection (ATCC). WEHI-3 cells were cultured and maintained in Dulbecco's Modified Eagle Medium (DMEM) supplemented with 10% heat-inactivated equine serum (Hyclone), 100 μg/ml penicillin/streptomycin and L-glutamine. The cells were incubated at 37°C in 5% CO_2_. All mice were maintained at Chinese PLA General Hospital (CPGH) in specific pathogen-free conditions. All animal experiments were performed in accordance with national and institutional guidelines for animal care and approved by the animal use and care committee, CPGH.

### DAC combined with cytarabine and lymphocyte infusion for mice with AML

Balb/c mice were injected *i.p*. with WEHI-3 cells (10^5^/mouse) and then randomly assigned into three groups. Group one was treated with PBS. Group two and Group three were injected *i.p*. with DAC (0.5mg/kg/d) and cytarabine (10mg/kg/d) for 5 consecutive days. For group three, splenocytes from naïve C57×Balb/c F1 (CB6F1) mice were injected *i.v*. at a dose of 4×10^7^/mouse 36 hours after DAC treatment. The survival of mice was observed every 2 or 3 days.

### Adoptive transfer of CD3^+^ splenocytes from DAC treated mice

For leukemia establishment, Balb/c mice were injected *i.p*. with WEHI-3 cells (10^5^/mouse). The mice were injected with either PBS or DAC (0.5mg/kg/d) *i.p*. once per day for 5 days. Treated mice were sacrificed seven days later for lymphocyte harvesting. Following red blood cell lysis procedure, splenocytes were labeled with PE-conjugated anti-mouse CD3 mAb and consequently anti-PE microbeads (Miltenyi), followed by MACS selection. Purity of positively selected spleen CD3^+^ cells was determined by FACS analysis. Purified CD3 positive cells (2×10^7^) from PBS or DAC treated mice were injected *i.v*. into Balb/C nude mice, which were injected with/without DAC-treated (1 uM for three days in culture) WEHI-3 cells (5×10^4^ cells/mouse) two days earlier. The survival of mice was observed every 2 or 3 days.

### Statistical analysis

SAS 9.0 software (SAS Institute) was used in all statistical analysis. Survival data were analyzed by the log-rank test, and survival curves were plotted with the Kaplan-Meier method. *t* test was used to assess the probability of significant differences of survival time. *P* < 0.05 was considered statistically significant.
